# Current Status of Knowledge about Cardiopulmonary Resuscitation among the University Students in the Northern Region of Saudi Arabia

**DOI:** 10.1155/2018/3687472

**Published:** 2018-06-10

**Authors:** Abdulmajeed Owaid Alsharari, Abdulrahaman Alduraywish, Ekremah Ali Al-Zarea, Naif Ibrahim Salmon, Md Sayed Ali Sheikh

**Affiliations:** Department of Internal Medicine, College of Medicine, Jouf University, Sakaka, Aljouf, Saudi Arabia

## Abstract

**Background:**

Sudden cardiac arrest is a major public health problem in the world. Immediate initiation of high-quality cardiopulmonary resuscitation (CPR) significantly increased patient survival rate. Therefore, it is very important to train young people and increase public awareness of CPR for the long-term benefit of the community.

**Objective:**

We aimed at estimating the level of knowledge and attitude towards cardiopulmonary resuscitation (CPR) among the university students in the northern region of Saudi Arabia.

**Methodology:**

A cross-sectional, prospective study was conducted among the students of four northern region universities of Saudi Arabia (Jouf, Hail, Northern Borders, and Tabuk) between March and November 2017. A self-administered questionnaire was prepared in both Arabic and English languages and distributed to all the participants. All the data were collected and analyzed by using SPSS version 21.

**Results:**

A total of 947 students from four universities completed the questionnaire: Jouf (57%), Hail (15%), Northern Borders (13%), and Tabuk (15%). Although 72% of students have previous knowledge about CPR, 49% of them lack knowledge about a medical emergency. Moreover, 59% failed to answer regarding CPR where only 41% wrote the ABC steps in the correct sequence. However, 67% of the participants had very poor knowledge, 89% of participants desired to receive additional CPR training course, and 49% of the students thought that CPR training should be a mandatory graduation requirement for all universities. There were no significant differences between male and female students. Students from medicine-related colleges have significantly (*p* < 0.001) more knowledge and scored better compared with non-medicine-related colleges. Tabuk University scored better compared to the others, but the overall knowledge and attitude scored were low.

**Conclusions:**

Overall knowledge about CPR among the university students was not satisfactory; however, attitude towards CRP training was very positive. Our results suggested that there is a need for improvement of CPR education among Saudi university students, which will help to reduce the cardiac arrest mortality rate among the community.

## 1. Introduction

**Figure 1:**
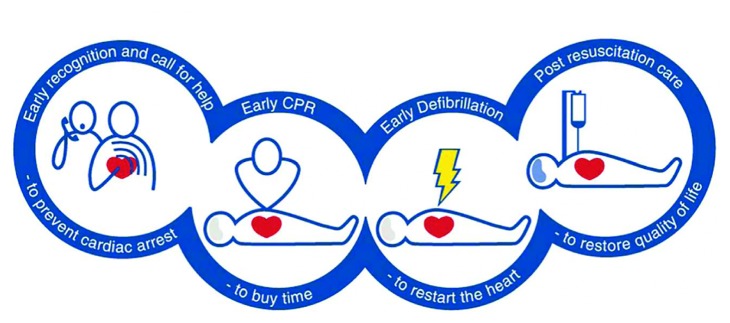
Chain of survival.

Cardiopulmonary resuscitation (CPR) has become a life-saving technique and is no longer limited to health-related professionals, which can be effective to decrease the mortality and morbidity in many medical emergencies such as heart attack, drowning, electrocution injuries, suffocation, and other conditions where the circulatory collapses. Sudden cardiac death (SCD) is a leading cause in 15%–20% of all deaths worldwide [1, 2] in which out-of-hospital sudden cardiac death responsible for more than 60% of all deaths among cardiovascular disease is the major cause of mortality worldwide [3–6]. One of the major issues with SCD is that most of out-of-hospital cardiac arrests (OHCAs) occur in patients as first clinical presentation of the underlying disease or who is already identified but categorized as low risk [2]. About 250,000 to 300,000 patients worldwide experience an OHCA per year [7]. However, survival rates among OHCA patients who receive bystander-initiated cardiopulmonary resuscitation (CPR) have a better chance of survival than those who do not [7]. Approximately 350,000 people in Europe die each year because of OHCA [7]. In the UK, approximately 60,000 people die of OHCA [8], and this number is predicted to be 300,000 and the mortality rate is 92% in the US [9, 10]. The number of SCDs in China is approximately 544,000 per year [11]. In Saudi Arabia, cardiac arrests show low survival rates [12], also in the Emirates of Dubai [13, 14]. Coronary heart disease (CHD) is the cause of SCD in more than 75% in the Western world and more than 50% in Japan followed by cardiomyopathies (<10% in the Western countries and <30% in Japan), inherited arrhythmia syndromes (10% in Japan and much lesser (<2%) in the Western countries), and valvular heart disease (<5%) [15]. Various studies have shown that 3%–12% of all acute myocardial infarction (MI) cases develop ventricular fibrillation (VF) resulting in SCD, and as many are found to be dead, the number is believed to be higher [16–20]. Nevertheless, ventricular fibrillation (VF) as a cause of out-of-hospital cardiac arrest [19] is low, and VF is still the most common underlying ventricular arrhythmia in SCDs [5, 21–23]. Techniques of CPR are aimed at maintaining the oxygenated blood flowing to vital organs, especially the brain, which is very sensitive to the absence of oxygen that can cause permanent brain damage within few minutes, and death may occur in less than 10 minutes [24]. Every minute after the onset of sudden cardiac arrest will decrease the benefit of resuscitation by up to 10% [25]. According to the 2015 American Heart Association guidelines [26, 27], the chain of survival (Figure 1) composed of five links: early access, immediate high-quality CPR, rapid defibrillation, basic and advanced emergency medical services, and advanced life support and postcardiac arrest care. In Turkey, a survey showed out of 533 people, only 3.6% can perform bystander CPR, and 15.6% answered the right compression-ventilation rate [28]. In a very recent study targeting the general dentists in Kuwait, 64% showed low knowledge, while only 36% have high knowledge about CPR [29]. In Saudi Arabia, few studies about CPR knowledge were conducted in a study on 753 subjects in Al-Khobar city in which 80.8% of the females and 86.5% of the males were totally unaware of CPR; however, 88.7% of all participants were willing to attend CPR courses in the future [30]. In another study conducted among secondary schools in Riyadh, 56% of the students did not have enough knowledge about CPR, and about 67% of all students wanted to know more about CPR [31]. A study at Qassim University that targeted health care professionals showed that 75% did not know what must be done after finding an unresponsive adult which requires activating EMS immediately [32], whereas 90.8% of medical and nursing students at Tabuk University did not know about activating EMS immediately [33].

Moreover, a survey performed in 2,250 university students in Riyadh showed 31% of the students have no knowledge about CPR and 85% of the students who have prior knowledge about CPR felt that it is insufficient [34]. This study aimed at estimating the level of knowledge and attitude towards cardiopulmonary resuscitation among the northern region university (Al-Jouf, Hail, Northern Borders, and Tabuk) students in Saudi Arabia. In addition, this study also aimed at determining their willingness to participate in CPR training in the near future.

## 2. Materials and Methods

### 2.1. Study Design

A cross-sectional, prospective study was carried out among four northern region universities (Jouf, Hail, Northern Borders, and Tabuk) of Saudi Arabia between March and November 2017.

### 2.2. Criteria of the Study Participants

The study populations included both male and female university students, aged between 18 and 29 years. They were physically and mentally sound and were willing to join our survey and also gave their spontaneous opinion regarding the knowledge of CPR. The participants were subdivided into two main groups: health-related college group (these college students are directly related to health science, e.g., school of medicine and hospital pharmacy) and non-health-related college group (these college students are not related to health science, e.g., college of law and college of engineering). We used a simple randomized sampling method for the selection of colleges.

### 2.3. Data Collection

We used a self-administered, structured questionnaire as previously described in Arabic language with minor modification [34]. The questionnaire consisted of (1) demographic data including age, gender, nationality, colleges, and academic years and (2) 14 questions which can be divided as of three sections: the first section (6 questions) assessed the participants' general CPR knowledge including source of the knowledge, the second section (6 questions) concerning about the attitude towards CPR included an ABC sequence of CPR, and the last section (2 questions) assessed the impression of participants and their suggestions to increase the awareness of CPR among the population. To determine the validity of our questionnaire, we randomly distributed a pilot survey among 20 students, but we excluded them from the main sample of this study. Several colleges from different universities participated in the study, and 947 questionnaires were filled properly, with a response rate of 91%.

### 2.4. Data Processing and Analysis

The data were analyzed statistically by using Statistical Package for Social Sciences (SPSS) version 21. The descriptive, inferential statistical analyses, along with comparative statistical analyses, were performed, and noncontinuous data were analyzed using the chi-square tests, and the *p* value was used to detect the significance of the parameters on a categorical scale between the groups when needed.

## 3. Results

**Figure 2:**
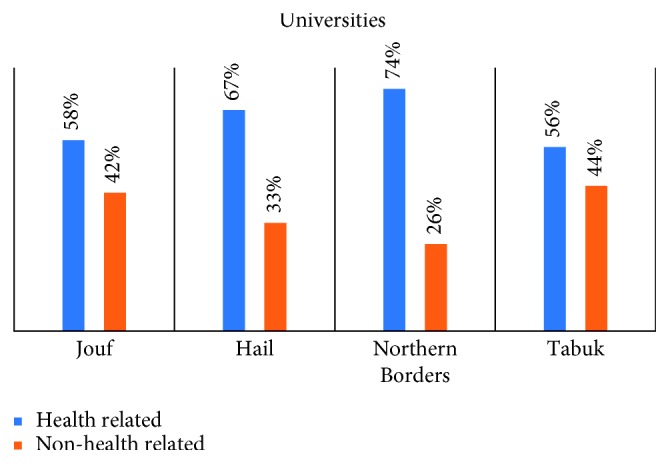
CPR knowledge between health- and non-health-related colleges for each university.

This study was carried out on 947 subjects who completed the survey, with a response rate of 91%. The demographic data are shown in (Table 1). The participants were all of Saudi nationality: 58% males and 42% females. The mean age was 21.55 years. We divided the sample into health-related colleges 578 (61%) and non-health-related colleges 369 (39%) (Figure 2).

**Figure 3:**
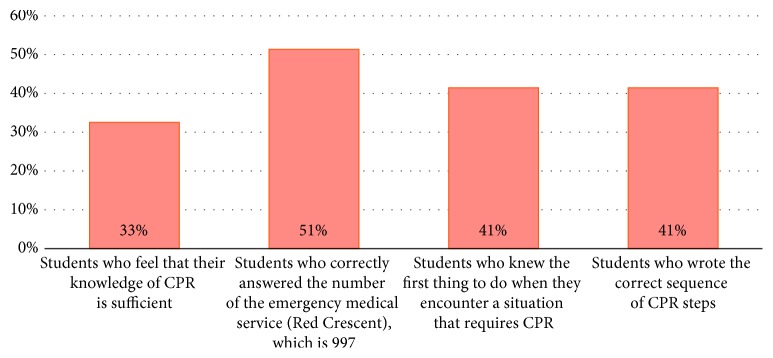
Students who have knowledge about cardiopulmonary resuscitation.

**Figure 4:**
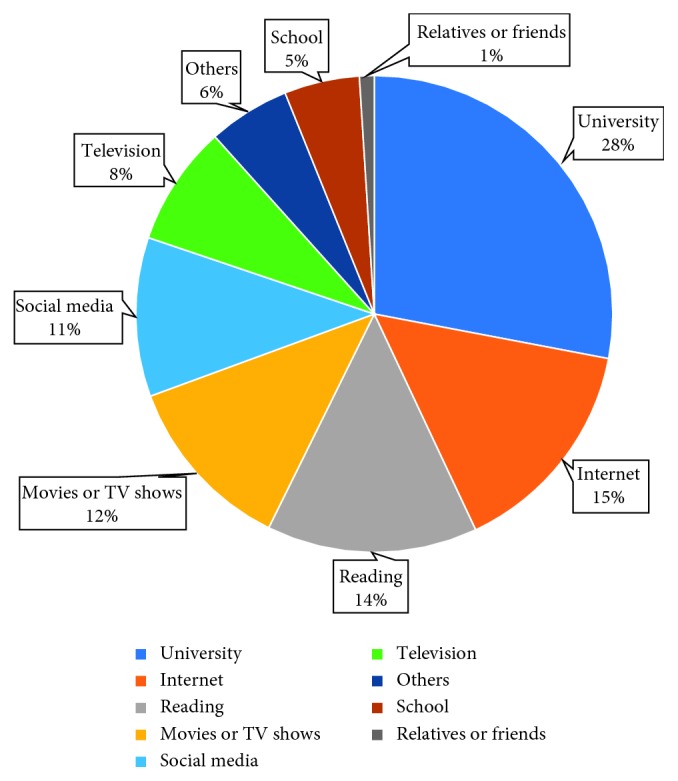
The sources of CPR knowledge.

The first section of our questionnaire (Table 2) result showed that 72% of the students have a previous knowledge about CPR, 67% of them think that it is insufficient, 51% of them correctly answered the number of the emergency medical service (Red Crescent), and 59% did not know what do they do first when they encounter a situation that requires CPR, whereas only 41% wrote the steps of CPR in the correct sequence (Table 3 and Figure 3). Interestingly, 89% of those who have no knowledge about CPR have willingness to take a CPR training course. The universities were the most common source of CPR knowledge for students by 28%, followed by the Internet (15%) and reading (14%) (Table 4 and Figure 4). Furthermore, 29% of the sample has been faced with a situation that needs CPR intervention where 51% of them preformed CPR and 28% did not perform it due to lack of knowledge.

The second sections of our questionnaires were specially focused on the attitude of the participants towards CPR (Table 5); we found that only 18% of the students have been taken a CPR training course. Among all participants, 60% of them were unable to write the correct sequence of CPR steps, and only 43% of them know the first step of the CPR. However, the majority of the students (90%) were willing to take a CPR course.

**Figure 5:**
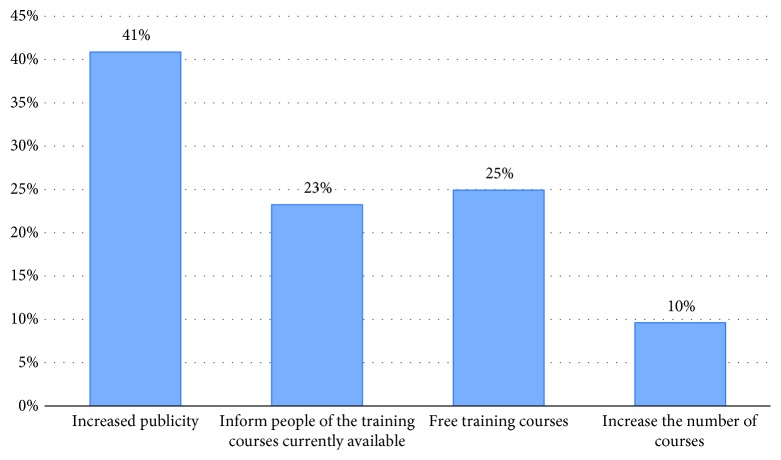
The effective method in students' opinion to increase public awareness about CPR.

**Figure 6:**
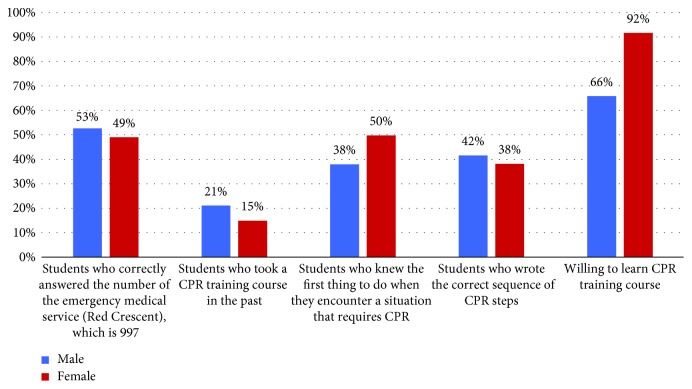
Comparison between both genders.

**Figure 7:**
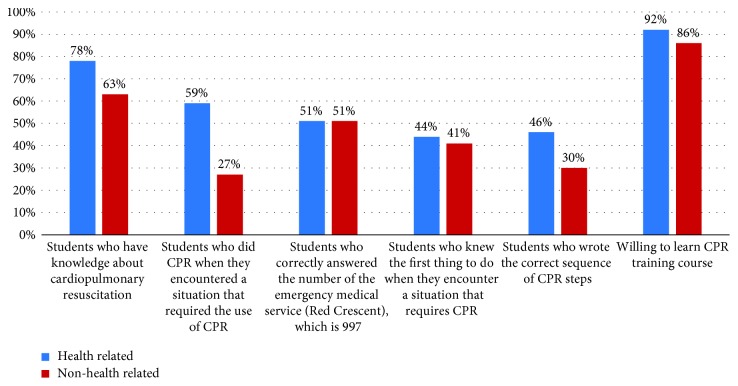
Comparison between health-related colleges and non-health-related colleges.

**Figure 8:**
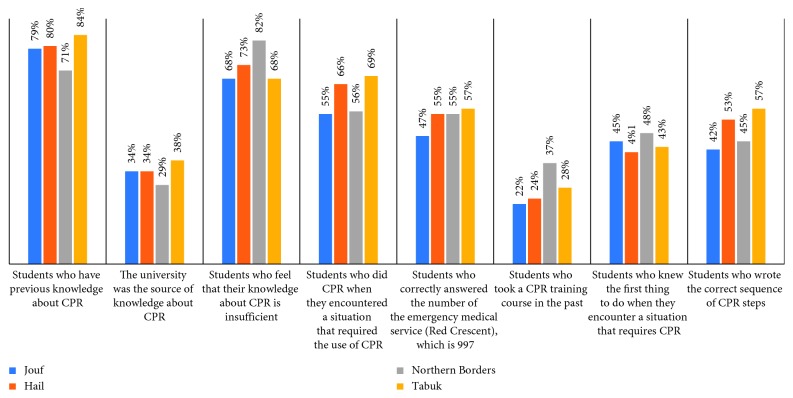
Comparison between health-related colleges of each university.

The last section of our questionnaire revealed that among all interviewed students, 49% think that CPR training should be mandatory for all the university students (graduation requirement), and 37% think that it should be mandatory for some majors, whereas 12% believe that it should be optional. Students were asked which methods were more efficient to increase community awareness about CPR (Table 6), and 41% believe that the media was the most effective and 23% agreed that informing people of the training courses currently available can increase awareness in the community. In addition, 25% recommended offering training course free of cost (Figure 5). We have made a comparison between both genders (Table 7 and Figure 6), and the result showed that there were no statistically significant differences between males and females about the CPR training course and medical emergency. We divided the samples into two groups, one group included the students from health-related colleges (e.g., medical, dental, pharmacy, nurse, and applied medical sciences) which consisted of 578 students (61%), and the other group from non-health-related colleges consisted of 369 students (39%). We have compared the two groups (Table 8 and Figure 7) and found that the health-related colleges have significantly more knowledge than non-health-related colleges. Furthermore, we also compared the knowledge regarding CPR knowledge among the health-related colleges of the four universities, and we found that Tabuk University had better scores compared with others (Table 9). Moreover, on average Jouf University students showed poor knowledge in most of the questionnaire points than other university students (Figure 8).

The role of the university was not quite enough as a source of knowledge about CPR in all four universities. However, less than half in each university knew that call for help is the first thing to do when they encounter a situation that requires CPR, and almost similar result was obtained when we asked them the correct CPR sequence, which seems to be as a result of not attending CPR courses. In fact, most of them fully agreed that their knowledge of CPR was insufficient.

## 4. Discussion

In this study, we tried to assess the community awareness by examining the knowledge about CPR among the students of the universities of the north region, which are Jouf, Hail, Northern Borders, and Tabuk. Although 72% of students have a previous knowledge about basic life support (BLS), they have a low level of basic knowledge and skills as more than a half of them do not know Red Crescent which is the first step of CPR; if we compare it with students at King Saud University, 70% know Red Crescent [34], and only 41% know the ABC sequence correctly; in fact, 67% of them think it is not enough and need more knowledge. On the contrary, about 89% of them who have no knowledge about CPR were willing to increase their knowledge by taking a CPR training course which is a positive view that must be exploited to increase the public awareness. Though it may seem obvious that the universities were the main source of CPR knowledge as all the samples were taken from them, they are still a part of the community; if it was not for the university, they would never have got that knowledge which indicated lack of other sources, including the role of mass media in disseminating the health messages to all the communities. Lack of knowledge about the first aid skills was the reason for 28% of students not performing CPR; when we compare it to the finding of similar studies [34], it was responsible for approximately a half of the situations where CPR was needed. However, it was responsible for 9.1% not performing CPR in the US study [35], in which both genders were willing to attend a CPR training course whenever possible. Previous studies in Saudi Arabia reported that 59% of females were not performing CPR compared to 29% of males [30]. In general, male's students have more knowledge about CPR as compared with female students; the reason is male students are more interested to do outdoor activities. Approximately all the participants of our study agreed that CPR knowledge should be an admission criterion by the universities either as mandatory or optional for all the majors. In a recent study in Nigeria, about 86% of students supported mandatory BLS training in school [36], whereas in Saudi Arabia, 69.5% think that the public awareness about CPR should be improved [33]. When we compared health-related colleges and non-health-related colleges, we expected that the first group will be better than the other, but it has to be more than our results showed for health-related colleges as they will work in places where this skill will be needed one day in their life. Tabuk University students had the highest knowledge, whereas Al-Jouf University students had the lowest one. In general, the low levels of knowledge were seen in all four universities in their health-related colleges. Other studies also in health-care professionals showed low level of CPR knowledge [37, 38], and in a similar study in Tabuk University, the knowledge was insufficient, and they suggested that BLS course should be included in course curriculum every year for the undergraduate students along with a regular reassessment which we strongly agree [33]. However, more importantly, few students of each university had a previous training in CPR course. A study found that a BLS course within the previous six months can significantly improve the knowledge and skills [39]. We recommended that all the universities should arrange a 3-week CPR training course in the final year and include it as a graduation requirement for the students. However, of course media can also play a vital role in increasing the public awareness of CPR.

In conclusion, the overall awareness about CPR was good, but some knowledge and first aid skills must be improved. More studies targeting the community to estimate the knowledge and attitudes should be carried out.

## Figures and Tables

**Table 1:** The characteristics and demographic features of the participants.

Variables	Frequency	Percentage
Age (mean ± SD)	21.55 ± 2.596	—
Gender (male/female)	551/396	58/42
Nationality (Saudi/non-Saudi)	947/0	100/0
Universities (health related/non-health related)		
Jouf (312/226)	538	57
Hail (96/47)	143	15
Northern Borders (89/32)	121	13
Tabuk (81/64)	145	15
Health related/non-health related	578/369	61/39

**Table 2:** Knowledge of the participants about cardiopulmonary resuscitation (CPR).

Variables	Frequency (%), *n*=947	
Do you have any knowledge about cardiopulmonary resuscitation?	Yes, 685 (72%)	No, 262 (28%)
Have you ever encountered a situation that required the use of CPR?	Yes, 271 (29%)	No, 676 (71%)
Do you feel that your knowledge of CPR is sufficient?	Yes, 238 (25%)	No, 709 (75%)
Do you call the EMS number in a medical emergency (Red Crescent)?	Correct, 484 (51%)	Incorrect, 463 (49%)

**Table 3:** Estimating the knowledge about CPR.

Students who have knowledge about cardiopulmonary resuscitation (*n*=685)	*n* (%)
Students who feel that their knowledge of CPR is sufficient	223 (33%)
Students who correctly answered the number of the emergency medical service (Red Crescent), which is 997	352 (51%)
Students who knew the first thing to do when they encounter a situation that requires CPR	284 (41%)
Students who wrote the correct sequence of CPR steps	284 (41%)

**Table 4:** Students' resources of CPR knowledge.

Students who have previous knowledge about CPR (*n*=685)	*n* (%)
University	192 (28)
Internet	103 (15)
Reading	98 (14)
Movies or TV shows	83 (12)
Social media	74 (11)
Television	56 (8)
Others	38 (6)
School	35 (5)
Relatives or friends	6 (1)

**Table 5:** The attitude of the participants towards CPR.

Variables	Frequency (%)	
Have you ever taken a CPR training course?	Yes, 175 (18%)	No, 772 (82%)
What do you do *first* when you encounter a situation that requires CPR?	Correct, 406 (43%)	Incorrect, 541 (57%)
Write the correct sequence of CPR steps	Correct, 380 (40%)	Incorrect, 567 (60%)
Do you want to learn CPR?	Yes, 848 (90%)	No, 99 (10%)

**Table 6:** The effective method in students' opinion to increase public awareness about CPR.

Increased publicity	387 (41)
Inform people of the training courses currently available	220 (23)
Free training courses	236 (25)
Increase the number of courses	91 (10)
Others	13 (1)

**Table 7:** Comparison between both genders.

Variables	Gender		*p* value
	Male, *n*=551	Female, *n*=396	
Students who correctly answered the number of the emergency medical service (Red Crescent), which is 997	290 (53%)	194 (49%)	*p* > 0.05
Students who took a CPR training course in the past	116 (21%)	59 (15%)	*p* > 0.05
Students who knew the first thing to do when they encounter a situation that requires CPR	209 (38)	197 (50)	*p* < 0.05
Students who wrote the correct sequence of CPR steps	229 (42)	151 (38)	*p* > 0.05
Willing to learn CPR training course	485 (66)	363 (92)	*p* < 0.05

**Table 8:** Comparison between health-related colleges and non-health-related colleges.

Variables	Health-related colleges	Non-health-related colleges	*p* value
Students who have knowledge about cardiopulmonary resuscitation	453 (78)	232 (63)	*p* < 0.05
Students who did CPR when they encountered a situation that required CPR	117 (59)	20 (27)	*p* < 0.05
Students who correctly answered the number of the emergency medical service (Red Crescent), which is 997	294 (51)	179 (51)	*p* > 0.05
Students who knew the first thing to do when they encounter a situation that requires CPR	256 (44)	150 (41)	*p* > 0.05
Students who wrote the correct sequence of CPR steps	268 (46)	112 (30)	*p* < 0.05
Willing to learn CPR training course	529 (92)	319 (86)	*p* < 0.05

**Table 9:** Comparison between health-related colleges of each university.

Variables	Jouf	Hail	Northern Borders	Tabuk
Students who have previous knowledge about CPR	245 (79)	77 (80)	63 (71)	68 (84)
The university was the source of your knowledge about CPR	84 (34)	26 (34)	18 (29)	26 (38)
Students who feel that their knowledge about CPR is insufficient	213 (68)	70 (73)	73 (82)	55 (68)
Students who did CPR when they encountered a situation that required the use of CPR	56 (55)	21 (66)	18 (56)	22 (69)
Students who correctly answered the number of the emergency medical service (Red Crescent), which is 997	146 (47)	53 (55)	49 (55)	46 (57)
Students who took a CPR training course in the past	68 (22)	23 (24)	33 (37)	23 (28)
Students who knew the first thing to do when they encounter a situation that requires CPR	139 (45)	39 (41)	43 (48)	35 (43)
Students who wrote the correct sequence of CPR steps	131 (42)	51 (53)	40 (45)	46 (57)
